# In vivo T2 measurements of the fetal brain using single-shot fast spin echo sequences

**DOI:** 10.1002/mrm.30094

**Published:** 2024-04-16

**Authors:** Suryava Bhattacharya, Anthony N. Price, Alena Uus, Helena S. Sousa, Massimo Marenzana, Kathleen Colford, Peter Murkin, Maggie Lee, Lucilio Cordero-Grande, Rui Pedro A. G. Teixeira, Shaihan J. Malik, Maria Deprez

**Affiliations:** 1 School of Biomedical Engineering and Imaging Sciences, King’s College London, London, UK; 2 Guy’s and St Thomas’ NHS Foundation Trust, London, UK; 3 Centre for the Developing Brain, King’s College London, London, UK; 4 Biomedical Image Technologies, ETSI Telecomunicración, Universidad Politécnica de Madrid and CIBER-BBN, Madrid, Spain

**Keywords:** dictionary fitting, fetal brain MRI, motion correction, relaxometry

## Abstract

**Purpose:**

We propose a quantitative framework for motion-corrected T2 fetal brain measurements in vivo and validate the single-shot fast spin echo (SS-FSE) sequence to perform these measurements.

**Methods:**

Stacks of two-dimensional SS-FSE slices are acquired with different echo times (TE) and motion-corrected with slice-to-volume reconstruction (SVR). The quantitative T2 maps are obtained by a fit to a dictionary of simulated signals. The sequence is selected using simulated experiments on a numerical phantom and validated on a physical phantom scanned on a 1.5T system. In vivo quantitative T2 maps are obtained for five fetuses with gestational ages (GA) 21–35 weeks on the same 1.5T system.

**Results:**

The simulated experiments suggested that a TE of 400 ms combined with the clinically utilized TEs of 80 and 180 ms were most suitable for T2 measurements in the fetal brain. The validation on the physical phantom confirmed that the SS-FSE T2 measurements match the gold standard multi-echo spin echo measurements. We measured average T2s of around 200 and 280 ms in the fetal brain grey and white matter, respectively. This was slightly higher than fetal T2* and the neonatal T2 obtained from previous studies.

**Conclusion:**

The motion-corrected SS-FSE acquisitions with varying TEs offer a promising practical framework for quantitative T2 measurements of the moving fetus.

## 1 Introduction

During the fetal period, the brain undergoes rapid growth and development. Important maturation processes, such as neurogenesis, neuronal migration, tract formation and myelination, result in microstructural changes observable in MRI.^1,2^ The observed changes in MRI contrast over this period^1,3–6^ suggest that the measurement of relaxation parameters may provide new understanding and characterisation of typical and atypical fetal brain development.

### 1.1 Motion-corrected fetal MRI

MRI is commonly used in clinical practice to further characterize congenital abnormalities detected by ultasound.^1,7^ During MRI scanning, free movement of the fetus and maternal breathing disrupts the image acquisition, limiting the applicability of the standard sequences commonly used in adult MRI. Fetal T2-weighted clinical scans therefore primarily use the single-shot fast spin echo (SS-FSE) sequence,^8^ which allows acquisition of the whole two-dimensional (2D) slice after a single excitation, with duration below 1s, thus effectively freezing motion in time.^9^ Fast acquisition of individual slices results in minimal in-plane motion during acquisition, thus providing good structural information that can support qualitative clinical assessments of fetal brain.^1,7,10^ However, the resulting acquired stacks of slices suffer from inter-slice motion corruption, which disrupts three-dimensional (3D) visualization and analysis. To address this limitation, several such stacks of slices with different orientations are co-aligned using slice-to-volume registration (SVR)^9,11,12^ interleaved with super-resolution (SR) reconstruction^13–16^ to provide fully motion-corrected isotropic 3D fetal brain images. Recently, extensions that provide fully automated reconstruction and focus on other fetal anatomy and placenta have also been proposed.^17–21^

### 1.2 Relaxometry

Relaxometry, the measurement of tissue relaxation in MRI, allows a direct quantitative evaluation of tissue properties using MRI, unlike standard weighted clinical sequences which are primarily used qualitatively. There have been rapid advances in techniques to measure relaxation parameters due to improvements in scans and data processing.^22^ Spin-echo sequences are commonly used to measure T2 and can be accelerated with techniques such as Multi-Echo-Spin-Echo (MESE).^23^ However, these sequences still require relatively long acquisition times (e.g., several minutes), which makes them sensitive to motion. Other techniques, such as variable flip-angle^24^ and DESPOT methods^25^ have been developed as alternatives but these typically require multiple 3D scans, which are again very sensitive to motion. Therefore, none of these techniques can provide reliable fetal relaxometry due to the challenge of rapid and unpredictable fetal movement independent of maternal anatomy, along with maternal breathing motion.^5,6,26,27^

Unpredictable fetal motion and maternal breathing presents a serious challenge for relaxometry in fetus, therefore dedicated fast acquisition methods, typically 2D single-shot sequences, need to be employed to perform such measurements. The earliest method, a gradient echo based mono-point estimation of fetal brain T1,^28^ obtained acceptable measurements, and more recently modified Look–Locker inversion recovery based on balanced steady-state free precession sequences was proposed for fetal brain T1 mapping.^29^ Both of these studies were limited to a small number of 2D slices and no motion correction was attempted. The multidynamic multi-echo method^30^ was recently proposed to measure T1 and T2 relaxation times in fetal brain; however, the measurement was successful only in 23% of the scans, due to corruption by motion. Nevertheless, the study highlights potential clinical value of T2 relaxometry in fetal brain, and need for motion correction to improve success rate.

A multi-echo Echo-Planar Imaging (EPI) with in-plane motion correction was proposed for T2* estimation in fetal brain^5^ and a motion-corrected pipeline using SVR was proposed to reconstruct 3D volumetric T2* maps of fetal brain.^6^ Multiple studies have proposed in-vivo T2* relaxometry in placenta using multi-echo EPI,^31–35^ including motion correction using 3D volumetric deformable registration. Very recently, the deformable SVR^18^ in tandem with multi-echo EPI was proposed to generate 3D T2* measurements of placenta,^17^ fetal brain^36^ and lungs.^37^

Placental T2 measurement was first performed in murine placenta in vivo using MESE imaging.^38^ In human placenta T2 relaxometry was first proposed for placenta using spin-echo EPI sequences.^39–41^ To deal with placental motion due to maternal breathing, 3D volumetric deformable registration was proposed.^41^ However, so far, none of these T2 measurement techniques were applied to the fetal anatomy.

The standard clinical MRI sequence to visualize fetal anatomy in 2D is T2-weighted SS-FSE,^8^ due to its resistance to motion, high soft tissue contrast, and relatively high resolution. There is only one study on a static physical phantom that performed T2 measurements using this sequence.^42^ The authors performed validation of the T2 measurements using SS-FSE acquired with multiple echo times (TE) against MESE and spin-echo standard and also included reconstruction using SR technique. The study demonstrated repeatability, however, they reported differences between T2 measurements using the native SS-FSE images with MESE and spin-echo standard. This may be due to lack of modeling of the slice profile effects, as we observed in this paper.

In fetal brain, T2 measurements were so far only performed using multidynamic multi-echo sequence with no motion correction as mentioned above.^30^ To our knowledge, there have been no studies that proposed motion-corrected volumetric in vivo T2 mapping of the human fetus, that would be reliable in presence of fetal motion.

### 1.3 Contributions

In this study, we aim to perform an accurate T2 measurement in fetal brain in vivo, that is both high spatial resolution and robust to fetal motion and maternal breathing, to enable future large clinical studies. We propose a framework^43^ to measure in vivo T2 relaxation time in the fetal brain using SS-FSE sequences with multiple echo times and SVR motion correction.^13^ High-resolution T2 maps are produced by fitting the images to a dictionary of signals simulated using extended phase graphs (EPG) that incorporate a slice profile correction. Similar methods for slice profile compensation are found in the literature.^44–47^ We validated the method without SVR on a numerical and a physical phantom and applied the entire framework, including SVR, to five in vivo fetal examinations as a demonstration of the framework and as a proof of concept. In vivo T2 values of fetal brain were measured, for the first time, for different fetal brain regions, namely the cortex, white matter (WM), deep grey matter (DGM), and subplate.

## 2 Methods

### 2.1 Quantitative T2 measurement framework for fetal brain

#### 2.1.1 Overview of the framework

**FIGURE 1:**
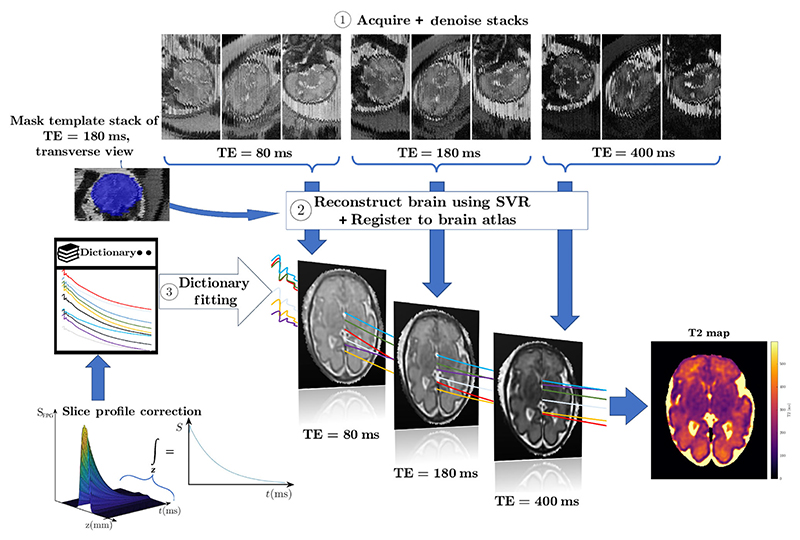
Diagram of the proposed pipeline.

The overview of the proposed T2 measurement framework for fetal brain is presented in Figure 1. First, we acquire SS-FSE stacks of fetal brain MRI with different echo times (TE) (Section 2.1.2, with three stacks with diverse views for each contrast (the selected views are given in Section 2.2.3). Next, the stacks are motion-corrected using SVR,^13^ producing a 3D volume for each TE. The 3D fetal brain images of different contrasts are then co-registered to ensure correct anatomical correspondences for voxel-wise fitting of the T2 values (Section 2.1.3). Quantitative T2 maps are calculated by fitting the acquired and motion-corrected MRI signals in each voxel using a dictionary of simulated signals^48–50^ (Sections 2.1.4 and 2.1.5).

#### 2.1.2 Sequence selection

We aim to build our T2 measurement framework with T2 weighted SS-FSE, which is a commonly used and clinically validated sequence for fetal anatomical imaging. We selected this sequence due to its excellent depiction of fetal brain anatomy. There is a trade-off between increasing the amount of data acquired (to improve T2 estimation) and maintaining an acceptable imaging duration. We decided to acquire a maximum of three different TE and explored an optimal selection of these for T2 estimation.

This study was performed on a Philip’s Ingenia 1.5T scanner at St. Thomas Hospital. Previous clinical studies by our institution suggest that the optimal TE at 1.5T for contrast in the fetal brain is around 180 ms, while the fetal body exhibits shorter T2 times, requiring echo times around 80 ms.^8^ Therefore, we opt to use these TEs to ensure that acquired images are also clinically useful. As we expect the fetal brain to contain water-rich transient WM structures such as the subplate and periventricular crossroads (resulting in long T2),^2^ but very little myelin or dense tissue (which would result in short T2), we choose a long TE of 400 ms for the third value. This is (approximately) the longest achievable TE within the bounds of our chosen sequence parameters, as detailed below.

To keep scanning time for each individual slice under one second, it is common to use partial Fourier and parallel imaging. To traverse the lines of k-space we select linear order, and vary the partial Fourier factor to achieve different echo times. Linear order starts at the truncated side of k-space and progresses through the center of k-space toward the highest frequencies. By varying the partial Fourier factor, we control the echo where the center of the k-space is acquired, thus effectively controlling the TE. This allows us to keep other acquisition parameters (e.g., bandwidth, echo spacing, resolution etc.) constant, keeping the image properties consistent for all echo-times. We obtain different contrast by varying the half-scan factor while keeping echo-spacing the same. We observed that this results in a constant point spread function due to the identical decay profile shifted in k-space for all of the different contrasts. The only difference between images is the partial Fourier artifacts. This, in turn, means that longer echo trains are used for the higher TE contrasts. The maximum TE in this setting will therefore correspond to the partial Fourier factor of ∼1, that is, almost a full sampling of the k-space. Other parameters were fixed and a summary of key parameters is shown in Table 1.

#### 2.1.3 Motion correction and 3D image reconstruction

The acquired data are corrupted by fetal and maternal motion primarily in the through-plane direction (shown in Figure 1). To correct this inter-slice motion, we use slice-to-volume reconstruction (SVR).^13^ This technique rigidly aligns each slice separately to an estimated 3D volume. After alignment, scattered data are reconstructed using SR techniques based on the inverse model of slice acquisition, and thus reduce the effect of blurring due to acquisition of thick slices (necessary for sufficient signal-to-noise ratio). Slices significantly affected by in-plane motion, or for which alignment failed, are removed using robust statistics. Differential bias fields and spin history effects are removed using intensity matching. The process is initialized by a volume reconstructed after volumetric rigid registration of all stacks to a template volume.

The template volume is important for SVR as its orientation and coordinate space defines the grid of the reconstructed isotropic 3D volume. In this work, we perform SVR for each contrast (TE) separately. In order to ensure that we obtain consistent anatomies for all the contrasts, we propose the following pipeline:

1. Denoise images using a Shearlet-based algorithm.^51^
2. Choose a template stack for one of the TEs (usually TE = 180 ms, unless it is deemed too motion corrupted upon visual inspection).
3. Manually mask the brain in the template stack using ITKSnap.^52^
4. Perform SVR^13^ for the selected TE, initialised by the masked stack, using the Slice-to-Volume Reconstruction ToolKit (SVRTK).^1^
5. Use this motion-corrected volume from the selected TE as a template volume to initialise the SVR of the remaining TEs.
6. Rigidly reorient all the reconstructed volumes to a standard space (defined by a structural fetal atlas^53^) using deep learning-based landmarks.^20^
7. Rigidly register the reconstructed and reoriented volumes for different TEs to the reoriented template volume to further align the anatomies, using the Medical Image Registration ToolKit (MIRTK)^2^

#### 2.1.4 Modeling of MRI signals to build a dictionary

The MRI signals of our proposed sequence (Section 2.1.2) exhibit complex evolutions due to non-ideal slice profiles and non-180° refocusing flip angles.^26^ Therefore, we propose to estimate the T2 maps from the motion-corrected multicontrast data (Section 2.1.3) using dictionary matching, in a similar manner to MR fingerprinting.^54^ To build the dictionary, we use the EPG formalism^49^ to model realistic MRI signals. To model the flip angle slice profile *θ*(*z*), where *z* is the spatial coordinate in though-plane direction, we use the radiofrequency (RF) pulse *B*_1_(*t*) and gradient waveforms *G*(*t*) to derive the resulting magnetization profile *M*_*z*_(*z, t*_end_) via Bloch equation simulation, where *t*_end_ is final time-point of the RF pulse. The flip angle is then derived according to Equation (1).

(1) $$\theta (z)=\text{arccos}\left(M_{z}\left(z,t_{\text{end}}\right)\right).$$

In the general case each pulse in the echo train may have a different RF waveform/amplitude. The corresponding flip angle profile *θ*_*i*_ is the profile of the *i*th pulse where *i* = 0 is the excitation pulse. Each pulse is obtained by performing a Bloch simulation separately, with the flip angle then defined as in Equation (1) where *t*_end_ is the time at the end of the pulse. The specific sequences we used contained constant refocusing pulses after the second refocusing pulse, that is, there were three different pulses to simulate: *θ*_0_, *θ*_1_ and *θ*_2 … *N*_.

These flip angle profiles along with T1, T2, and ESP, are used in the EPG formalism to generate the time-dependent signals *S*_EPG_ at each point *z* on the slice profile. The signals are integrated along the slice profile get the simulated MRI signals **S**(T2), as shown in Equation (2).

(2) $$\text{S}\left(\text{T}2\right)=\int_{z}{\text{S}}_{\text{EPG}}\left(\text{θ}\left(z\right),\text{T}2,\text{T}1,\text{ESP}\right)dz.$$

In Equation (2), *θ*(*z*) is a matrix of the flip angles as a function of space (*z*) and **S**_EPG_ is the matrix of signal intensities as a function of space (*z*) and for each RF pulse as a result of the EPG simulation. **S**(T2) is a vector of signal intensities for the *N* echoes as a result of the integration of **S**_EPG_ along its spatial points (*z*). In the EPG simulation, ESP and T1 are kept constant. Following this, *S*_TE_(T2) represents the *i*th element of the signal vector **S**(T2), where *i* is the index of the echo at which the center of k-space is encoded (i.e., the echo corresponding to the TE of the image). The dictionary is constructed from repeated simulation for different discrete T2 values and selecting the TEs needed for the images acquired. That means each entry of the dictionary is a vector of Signal intensities, the elements of which correspond to the TE of the images and, as such, these can be represented as **S**_T2_.

**FIGURE 2:**
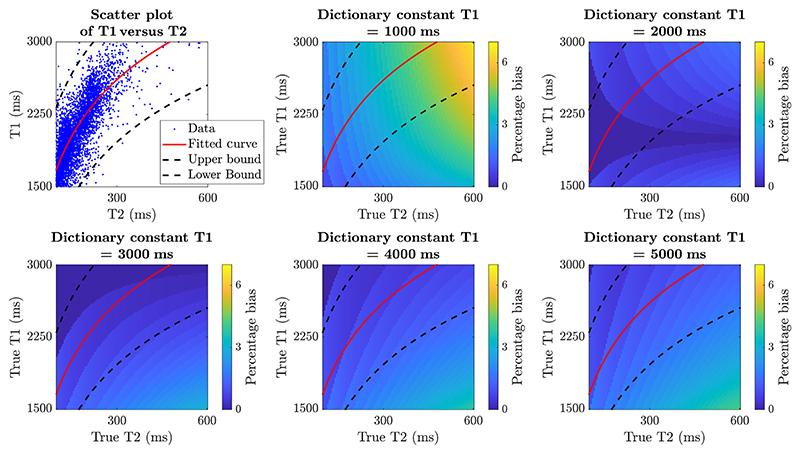
Investigation on a dictionary constant T1. The distribution of T1s and T2s in the neonatal maps is presented by the scatter plot on the top left. The pattern of bias caused by estimating T2 with fixed T1 is depicted by the rest of the plots.

Looking back at Equation (2), SS-FSE signals, in fact, depend on T1, but only weakly. Therefore, a fixed T1 value was considered appropriate to build the dictionary. The best fixed T1 value was investigated by simulating different T1 and T2 values. The difference between the predicted T2 from the dictionary (with constant T1) and the true T2 (from the simulated signals with true T1s) was measured, shown in Figure 2.

A scatter plot of T1 versus T2 values from the neonatal quantitative map (Figure 2 top left) shows the range of expected T1 and T2 values in this age range. The bounds were obtained by fitting a logarithmic trend line to the data and obtaining the 95% confidence intervals. In Figure 2 bottom left we can observe that for a constant parameter of T1 = 3000 ms the bias is within 2% between the upper and lower 95% confidence bounds. In addition, the trend line follows the area of lowest error in the color-map in this plot, for a potential broader range of T2 values in the fetus.

Therefore, we generate the dictionary of simulated signals for different T2 values while keeping T1 constant (T1 = 3000 ms). The resolution of the dictionary was 1ms for a range of T2 = [25, 3000] ms. The dictionary was generated in MATLAB using the open source EPG-X code^3^ for fast spin-echo (FSE, or Turbo spin-echo/TSE) sequences.^49,50^

#### 2.1.5 Estimating T2 maps using dictionary matching

We perform voxel-wise estimation of the T2 maps by fitting the motion-corrected MRI signals (Section 2.1.3) to the dictionary described in Section 2.1.4. Dictionary fitting is performed by finding the entry of the dictionary (T2 value) that results in the maximum scalar product between the dictionary entries and the measured signal intensities. Both the data and the dictionary are normalized by the Euclidean norm as shown in Equation (3). Here ***S***_T2_ is the vector of dictionary entries and $\bar{S_{i}}$ is the vector of the measured signal intensities for the *i*th voxel corresponding to all TEs. Maximization is performed on all T2 values in the dictionary.

(3) $$\hat{\text{T}2}=\text{arg}\;\underset{\text{T}2}{\text{max}}\frac{S_{\text{T}2}}{\left|S_{\text{T}2}\right|}\cdot \frac{{\bar{S}}_{i}}{\left|{\bar{S}}_{i}\right|}.$$

### 2.2 Experiments

The difficulty in validating the quantitative measurements in fetal brain is that there is no gold-standard sequence that could be used without being disrupted by fetal motion. We, therefore, perform a series of experiments to validate various aspects of the proposed quantitative framework (Section 2.1).

First, we validate our chosen sequence and parameter selection using a numerical phantom generated from real T1 and T2 maps of a neonatal brain, acquired using joint system relaxometry^55^ (Section 2.2.1). We further validate our sequence experimentally using a physical phantom, in comparison with the gold standard MESE sequence (Section 2.2.2). Finally, we acquire real fetal data using the proposed sequence, and compare the measured T2 values in fetal brain to neonatal T2 values and fetal T2* values from the literature (Section 2.2.3). The evaluation of the motion correction step (Section 2.1.3), which was found to preserve the accuracy of the T2 measurements, is presented in a separate publication.^56^

#### 2.2.1 Numerical phantom experiments

To model an in vivo fetal brain, a maternal body and amniotic fluid were artificially generated based on the literature T1 and T2 values (maternal body T1 = 1000 ms, T2 = 80 ms, and amniotic fluid T1 = 2800 ms and T2 = 2000 ms).^57^ In Figure 4, the circle represents the amniotic fluid and the ellipse represents the maternal body. A model fetus was added by taking the central slice from a neonatal relaxometry measurement,^55^ scaling the relaxation times to account for differences between fetus and neonate due to changes in water content. An approximate scale of a 40% increase in T2 and 20% increase in T1 was estimated from the literature.^5,6,29^ This formed a numerical fetal phantom for the set of simulated experiments performed.

A forward model was generated using the EPG simulation to generate simulated k-space data that was subsequently reconstructed using inverse FFT. The slice profile was also modeled as described in Section 2.1.4. The scanner parameters used were identical to those presented in Section 2.1.2. In these experiments, no acceleration (such as SENSE) or coil sensitivity profiles were modeled. However, we evaluated different options of the final TE (TE_3_ = [250,300, 350,400]), to conclude that the longest possible TE was necessary in the sequence.

Region of interest (ROI) analysis was done on cortical and DGM (combined as “grey matter” in the results) and WM of the numerical phantom. Segmentations were obtained using DrawEM.^58,59^

#### 2.2.2 Physical phantom experiments

Phantom validation experiments were performed using five vials of diluted MnCl_2_ (at 0.01, 0.05, 0.1, 0.15 and 0.2 mmol/dm^3^) and a spherical flask with 0.5% agarose gel (Figure 6). MESE measurements were also made, for comparison, and a dictionary of simulated signals from the MESE sequence, using EPG (with slice profile correction), was fitted to make the MESE T2 maps.

The phantom is non-moving and, therefore, only one view was taken per contrast (no motion correction needed). For the reference MESE scans, 3D and 2D sequences were used with FOV 150 × 150 mm, no SENSE factor, 15-mm 3D k-space encoding (using slab selective pulses) for the 3D MESE and 20 TEs at 20 ms spacings for all the MESE sequences. In addition to the dictionary fitting, an exponential decay model was used on the single-shot images and Equation (4) was minimized to obtain the T2 estimate.

(4) $$\text{T}2_{i}=\underset{\text{T}2,S_{0}}{\text{min}}∥S_{0}e^{-\text{TE}/\text{T}2}-S_{i}{∥}^{2}.$$

#### 2.2.3 Fetal measurements

In vivo fetal scans were acquired, using the parameters specified in Section 2.1.2. Based on the results of simulated experiments (Section 3.1), the maps used a TE combination of 80, 180, 400 ms. For each TE, three stacks with different orientations were acquired to enable motion correction (Section 2.1.3), nine stacks in total. In addition, it was important to ensure consistency of the scans. Therefore, RF power calibration and receiver gain adjustment was performed only once for the entire set of stacks and all nine stacks were acquired without any further radiological planning, in a predefined range of orientations with respect to maternal anatomy. The planes of the three different orientations were not exactly orthogonal because perfectly orthogonal views would cause the maternal anatomy to be outside the FOV and this would create wrap-around effects. Therefore, radiographers would adjust the view based on the individual anatomy within the predefined range. The radiographers aimed for one axial and two oblique sagittal views (between 35° and 45° from the midline).

This study had ethical approval (REC 07/H0707/105) and all participants who participated in this study consented to their scans being used for research. Five subjects were scanned at gestational ages 21, 27, 29, 31, and 35 weeks. The scans were added to a clinical protocol and the subjects were referred on the basis of the following developmental anomalies:

- GA 21 weeks: Moderate ventriculomegaly (VM)
- GA 27 weeks: Bilateral Borderline VM
- GA 29 weeks: Small brain for GA
- GA 31 weeks: Small hippocampus (HC)
- GA 35 weeks: Microcephaly/varicella exposure

**FIGURE 3:**
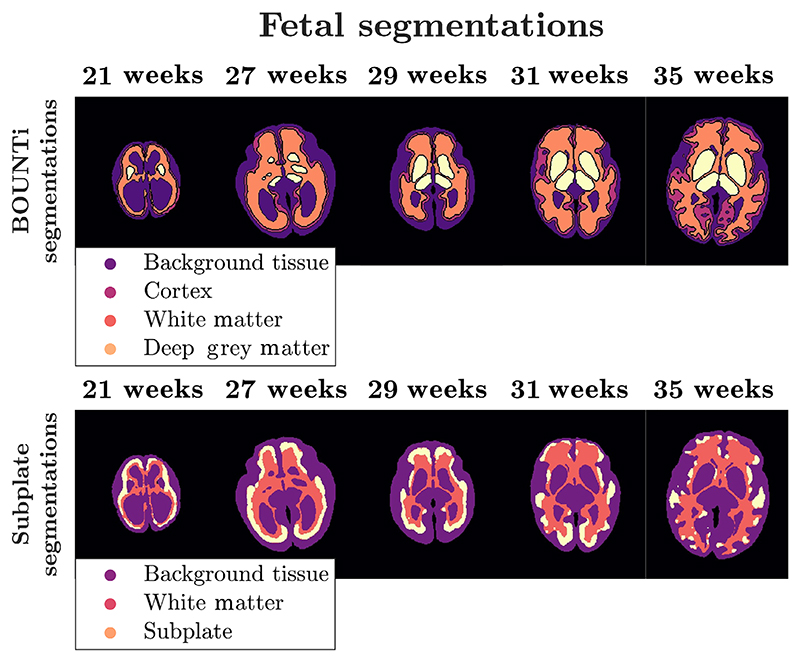
Segmentations for the Cortex, white matter and deep grey matter (top row) and the subplate (bottom row – white matter also displayed for anatomical reference).

Following reconstruction, five regions of interest (cortex, WM, DGM, and subplate) were segmented in the TE = 180 ms image using CNN-based segmentation.^60,61^ These segmentations were then eroded to avoid partial volume effects in analysis. All of the volumetric data are given in Table S1. Segmentations for the ROIs are shown in Figure 3.

## 3 RESULTS

### 3.1 Parameter and sequence optimizations

**FIGURE 4:**
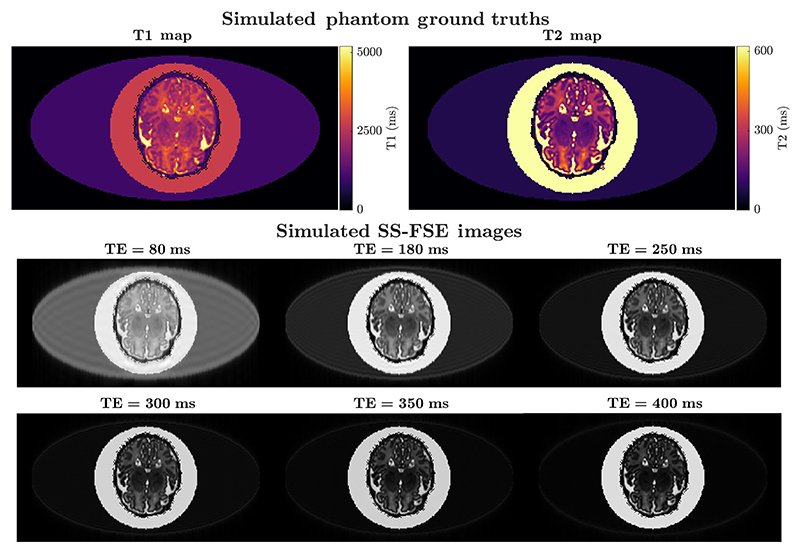
Ground truth maps from numerical phantom and the subsequent simulated images of the phantom. The eclipse shows the simulated maternal body and the circle is the simulated amniotic fluid.

The simulated MRI images from the numerical fetal phantom (Section 2.2.1) are shown in Figure 4. Ringing is very observable in the image of lowest echo time (the shortest echo time is obtained using a very significant partial Fourier factor—58% of k-space covered). For the highest echo time, there is still sufficient signal to be used to fit the model.

**FIGURE 5:**
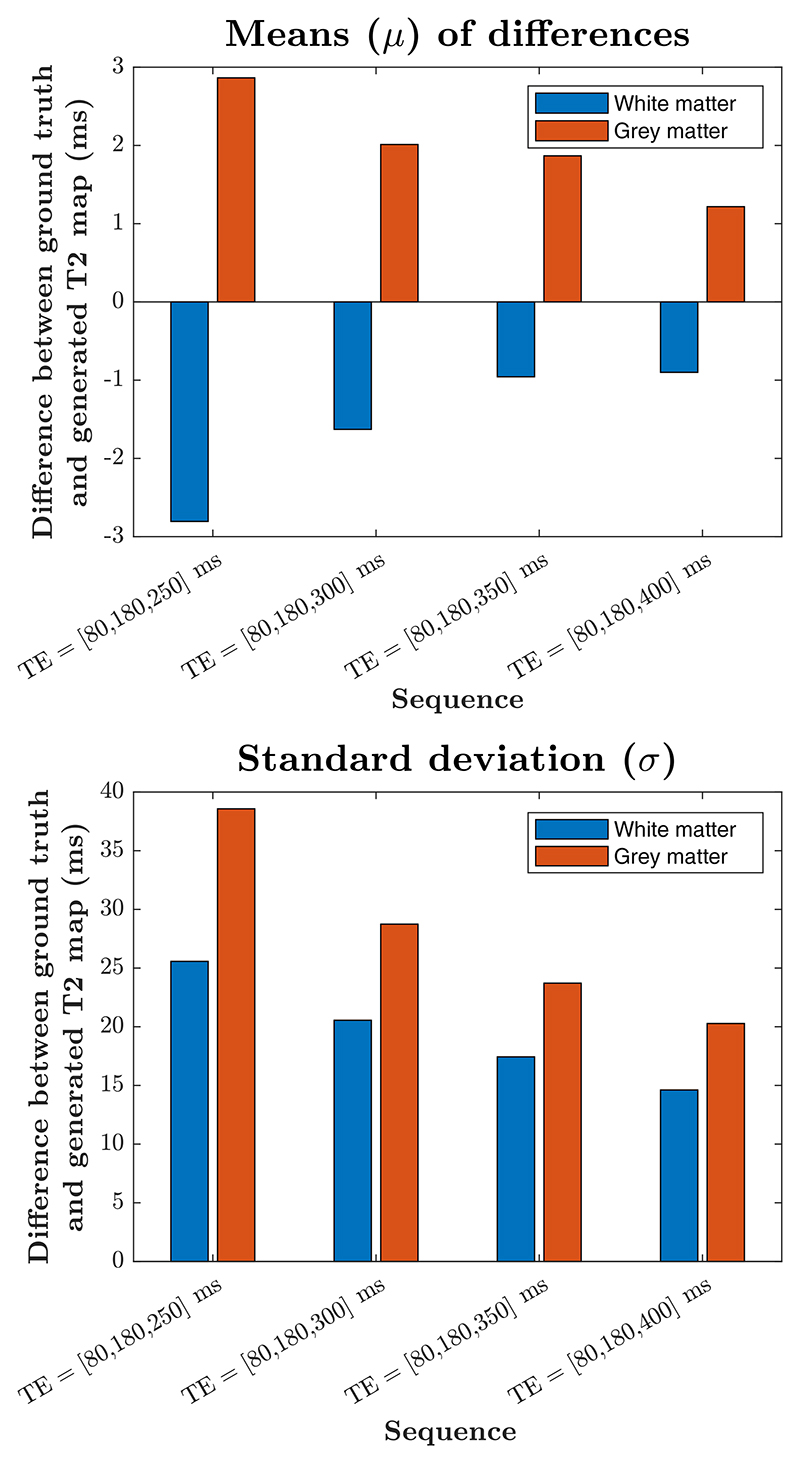
Summary statistics for differences between ground truth and predicted T2 maps obtained from the simulated experiments.

Figure 5 presents summary statistics (mean and standard deviation or SD) for differences between the ground truth and predicted T2 map within WM and GM regions. More detailed Bland–Altman plots are provided in Figure S1. The analysis suggests that the fit improves consistently as the third echo time increases. As 400 ms is the maximum achievable TE, which corresponds to the maximum sampling of the k-space, we select this value for the third TE of our proposed sequence. Importantly we can also observe that mean error is very small in all cases (within 3 ms), suggesting the proposed sequence produces unbiased measurements. SD of T2 = 20 ms for TE = 400 ms is also very low (less than 10% of the mean WM T2 of 216 ms and mean combined cortical and deep GM T2 of 129 ms).

In addition, we looked at TE_1_ and TE_2_ and the effects of changing these in the pipeline. However, in the experiments, we found that there was not a significant difference from the clinically suggested TEs of 80 and 180 ms and other nonclinically recommended echo times, given both mean difference (bias) and SD of differences (precision). This can be seen in Figure S2.

### 3.2 Physical phantom experiments

**FIGURE 6:**
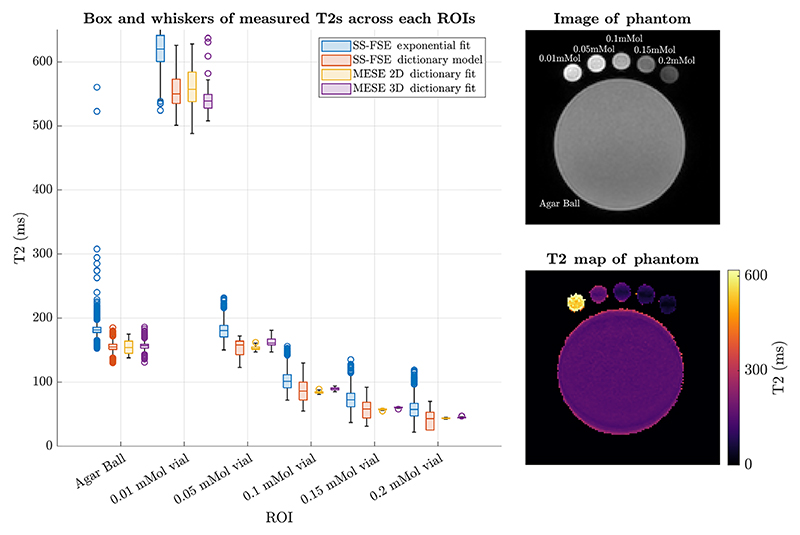
Comparing T2 distributions over different parts of phantom for the different measurement methods. The labeled image of the phantom (derived from one of the multi-echo spin echo images) shows the different regions being compared in the Box and Whiskers plot. The map is an example of the SS-FSE fitting using the Dictionary.

Figure 6 presents regional T2 values for the physical phantom, measured by our proposed sequence (Section 2.1.2) and gold standard MESE. The plot suggests good agreement when dictionary fit was used (Sections 2.1.4 and 2.1.5). Estimation of T2 maps with an exponential model leads to a systematic bias in the estimated T2, proving that the EPG based dictionary fit is essential for accurate T2 measurements.

The observed difference in T2 measurements between MESE and SS-FSE for short T2 can be explained by the long echo trains that are present in the SS-FSE sequences, in comparison to that of the MESE. SS-FSE was optimized for measuring longer T2 so is not as effective for very short T2 samples. We also performed repeatibility measurements on the same scanner using the same parameters. We observe in Figure S3, that there is a strong agreement between both scanning sessions. This is evident by comparing both the first set of scans and second set of scan sessions suggesting repeatability in the scanner that we used.

### 3.3 Fetal T2 measurements

**FIGURE 7:**
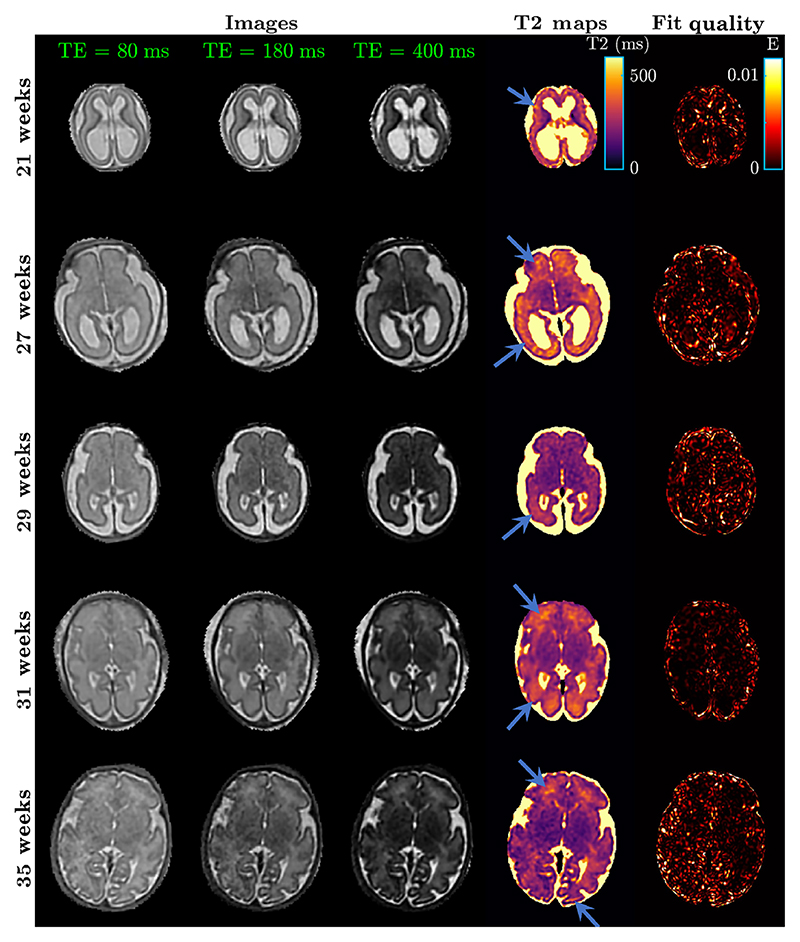
The reconstructed brain images, generated fetal maps and their fit quality maps. The fit quality was measured using Equation (5). Blue arrows point to subplate and periventricular crossroad regions.

Figure 7 shows the T2 maps of individual fetal subjects with their corresponding motion-corrected images for each echo time. We observe that the generally good delineation of anatomical structures in the source SS-FSE images carries through to the T2 maps. Anatomy is clearly distinguishable and certain features are emphasized in the T2 maps, such as the subplate and periventricular crossroad regions, displayed by the arrows in Figure 7.

In addition, we also display maps that demonstrate the quality of fit across the fetal brains (Figure 7, last column). Quality of fit is measured using Equation (5) for each voxel (based on Equation 3).

(5) $$E=1-\frac{S_{\text{T}2}}{\left|S_{\text{T}2}\right|}\cdot \frac{{\bar{S}}_{i}}{\left|{\bar{S}}_{i}\right|}.$$

This result suggests that the normalized dictionary signal entries fit the normalised voxel intensities really well, as the maximum value for any voxel of this map can be 1. The quality of the fit maps shows that mostly noise and partial volume cause any drops in fit quality. Figure S4 also shows a maximum solution to the scalar product, which suggests that there is a unique T2 value obtained through dictionary fitting.

**FIGURE 8:**
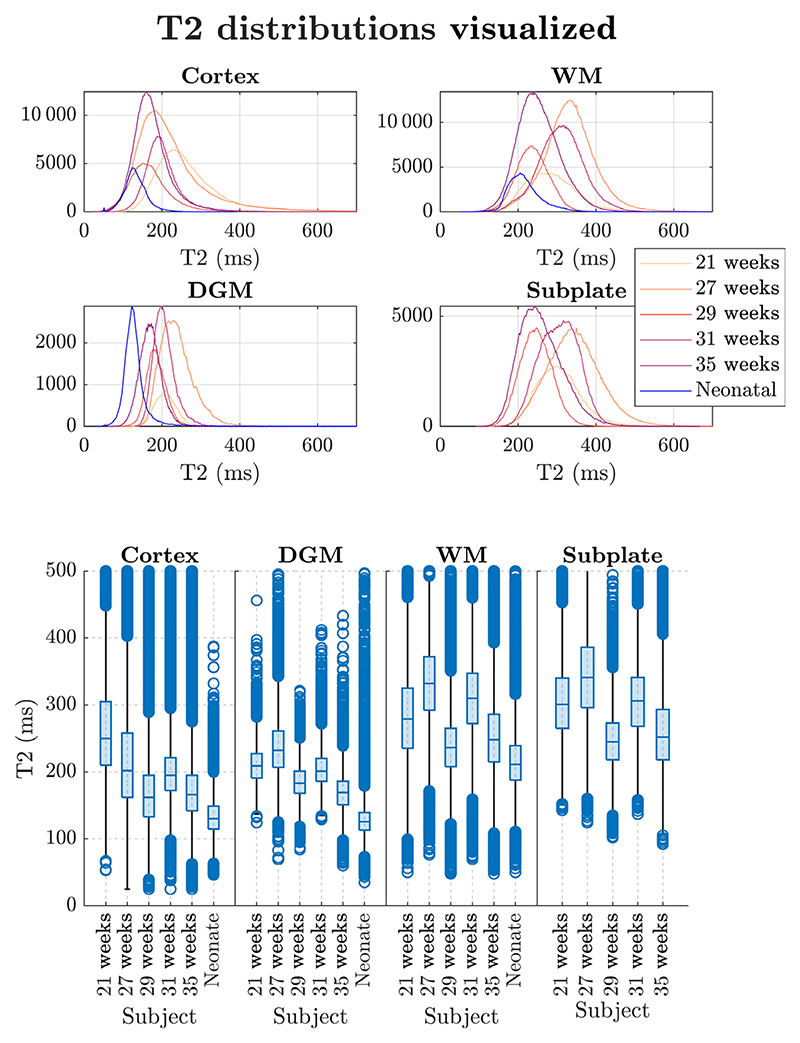
Distributions of T2 measurements for fetal subjects (with a comparison to the Neonatal subject) across different regions of interest.

The distributions of T2 values for different tissues in individual fetuses are presented in Figure 8. While these plots potentially suggest expected trends (e.g., decrease in T2 with GA), the number of subjects is not sufficient to derive definite conclusions. The subject with a GA of 29 weeks is an outlier with generally lower T2 values compared to the other subjects (except in the DGM where the oldest fetus has the lowest T2). This may be due to poor signal to noise ratio observed in this dataset, or other imaging limitations as radiological reports do not suggest any anomalies apart from a small head at GA. However, little is known about pathological changes in T2 of the fetal brain due to lack of suitable measurement technique to date.

Table 2 presents the average tissue-specific T2 values measured in the fetal brain of the five subjects included in this study. The ROI analyses were done based on the segmentations in Figure 3. We measured average T2 of approximately 200 ms in gray matter and just below 300ms in WM. These fetal values are consistently higher in comparison to the neonatal values, which is consistent with declining water content as the gestational age increases.^62^ Additionally, the measured T2 values are slightly higher than T2* values from the literature, also to be expected.

## 4 Discussion

Structural T2-weighted sequences such as SS-FSE are used routinely for qualitative clinical diagnosis,^8^ often coupled with SVR^9,11,12,14^ and SR^13,15,16^ for further volumetric assessment^8^ and biometric measurements.^63^ Our ability to reliably measure T2 relaxation time in fetal brain would allow us to identify subtle changes of tissue properties, facilitating more detailed assessment of fetal brain maturation processes, such as myelination, changing microstructure and water content,^30,64^ as well as pathology, such as presence of infection.^34,65^

Our proposed framework to obtain T2 measurements of the fetal brain combines the SS-FSE sequence with three different contrasts (with three views of different orientations for each contrast), motion correction using SVR and a EPG-based dictionary matching to estimate voxelwise T2 relaxation times. The pipeline was developed with the aid of simulated experiments using a numerical phantom, validated on a physical phantom and then used to perform measurements on five fetal subjects. The phantom validation experiments showed that SS-FSE with dictionary matching gives comparable T2 estimates to those obtained using more standard MESE approaches.

One limitation demonstrated in the physical phantom experiments was the low precision in the vials with a high concentration of MnCl^2^ (and, therefore, a low T2 of 50–80 ms). However, the measured fetal brain values tended to be higher than T2 = 100 ms with only a few voxel outliers of T2 < 100 ms. Therefore, this limitation is not likely to signicantly affect the precision of the T2 measurements in fetal brains.

While the phantom experiment does not validate the accuracy of the measurement when fetal motion is present, we have established in a separate publication^56^ that motion-correction using SVR^8,13^ does preserve the T2 values in simulated experiments performed using a modified Fabian simulator.^56,66^

There is a lack of existing fetal T2 measurements at the time of this study, and therefore we have compared our fetal brain T2 measurements to fetal T2* and neonatal T2 values. We expected a slightly higher fetal T2 than T2* and significantly higher fetal T2 than neonatal T2, both of which were observed in measurements made in this study.

Our image reconstruction relies on manual masking followed by standard SVR^13^ on the brain to reconstruct images in different contrasts. This results in good motion correction for the different images and good anatomical detail in the brain. However, this means that there is a requirement of nine extra scans for just three data points—having more data points would improve signal fitting. In addition, such separate reconstructions may potentially result in an increased need for data redundancy as well as reduced quality of alignment between individual contrasts. Future work is needed to address these limitations by developing end-to-end motion-correction and reconstruction of T2 maps by combining SVR and T2 fitting within an unified reconstruction model. This can also perhaps allow us to take stacks with unique orientations and unique TEs further increasing the number of datapoints on the signal model.

Additional future improvements might take advantage of deep learning to fully automate the reconstruction process^8,20^ and possibly require fewer acquired data. Such improvements may help shorten the acquisition and processing time, making this framework suitable for more extensive and, potentially, clinical use. This study was unable to identify clear trends in T2 of the fetal brain with respect to gestational age due to a limited number of subjects. In future, a larger cohort study may be able to establish such trends and thus provide insight into the biological changes in the developing fetal brain.

## 5 Conclusions

We developed a framework to measure T2 of the fetal brain in vivo. This framework includes image acquisition from a 1.5T system, SVR brain reconstructions for each TE and dictionary fitting to generate 3D T2 maps. We successfully validated the use of SS-FSE with dictionary fitting on numerical and physical phantoms. Following this, we implemented the entire framework with SVR on five in vivo fetuses to obtain initial T2 values for the cortex, WM, DGM and subplate. The validations suggests that SS-FSE and dictionary fitting result in minimal bias for T2 measurements and the fetal measurements suggest that fetal T2 is higher than fetal T2* and neonatal T2. We have therefore demonstrated a promising practical framework for quantitative T2 brain measurements of the moving fetus. Following this study, we hope to expand our implementation to a larger dataset to establish the trends in T2 values in the developing fetal brain.

## Supplementary Material

Supporting Information

## Figures and Tables

**Table 1:** The parameters for the different contrast images.

Variables	SS-FSE sequence parameters		
TE (ms)	80	180	400
TR (s)	17.8	21.4	29.1
ESP (ms)	6.2	6.1	6.1
Shot duration (ms)	510	600	824
Halfscan factor	0.587	0.709	0.966
TSE factor	82	99	135
FOV	350 × 350 × 100	350 × 350 × 100	350 × 350 × 100
Resolution (mm)	1.25 × 1.25 in plane	1.25 × 1.25 in plane	1.25 × 1.25 in plane
Slice thickness (mm)	2.5	2.5	2.5
Slice gap (mm)	−1.25	−1.25	−1.25
Number of slices	79	79	79
SENSE factor	2	2	2
WFS (pix)/BW (Hz)	0.496/437.7	0.496/437.7	0.496/437.7
Reconstruction matrix	288	288	288

*Notes*: These parameters included repetition time (TR), echo spacing (ESP), shot duration, halfscan factor, Turbo Spin Echo (TSE) factor or echo train length, field of view (FOV), resolution, slice thickness, slice gap, number of slices, SENSE acceleration factor, Water Fat Shift (WFS)/Bandwidth (BW) and reconstruction matrix.

Abbreviation: SS-FSE, single-shot fast spin echo.

**Table 2:** Table comparing T2 values with literature and neonatal values.

	Average fetal	Literature fetal	Literature fetal	Neonatal
Tissue	T2 (ms)	T2* (ms) ±*σ* (ms)	T2* (ms) ±*σ* (ms)	T2 (ms) ±*σ* (ms)
	±*σ* (ms)	(Vasylechko^5^ 1.5T)	(Blazejewska^6^ 1.5T)	using JSR^55^ on 3T
Cortex	199 ± 33	—	163 ± 30	133 ± 29
DGM	201 ± 26	Thalamus: 154 ± 24	—	132 ± 45
WM	283 ± 39	Frontal WM: 234 ± 38	Frontal WM: 259 ± 34	218 ± 50
Subplate	292 ± 40	—	268 ± 17	—

*Notes*: This table compares values of fetal T2 of this study with neonatal T2 and literature fetal T2*. A table including the mean values over each ROI (±*σ* over the ROI) for each subject is given in Table S2. Abbreviations: DGM, deep grey matter; WM, white matter.

Abbreviations: DGM, deep grey matter; WM, white matter.

## Footnotes

1 https://github.com/SVRTK/SVRTK.

2 https://github.com/BioMedIA/MIRTK.

3 https://github.com/mriphysics/EPG-X.
