# Supplementary material for: In vivo T2 measurements of the fetal brain using single-shot fast spin echo sequences
Source: Magn Reson Med. Author manuscript; Available in PMC 2025 Jan 7. (PMC7617281; doi:10.1002/mrm.30094)
Supplement: Supporting Information [file EMS201790-supplement-Supporting_Information.pdf]

SUPPORTING INFORMATION

The following supporting information is available as part of the online article:

Figure S1. Bland-Altman plots for the numerical phantom measurements to demonstrate the optimal sequences used. The top row is the Bland-Altman plots for the cortical and DGM and the bottom row is the WM.

Figure S2. Heatmaps showing the effects of all three TEs on the difference between the T2 values of ground and predicted map. TE_2 does not significantly impact the prediction, TE_1 slightly affects the mean difference (or bias) and TE_3 significantly affects both the mean differences and the standard deviation of differences.

Figure S3. Plots showing the comparison of T2 results from two sessions to assess repeatability of the measurements. These show repeated overlap with of all T2s; both the standard MESE and the proposed SS-FSE sequences.

Figure S4. Plots showing Scalar products for a voxel within the given ROI, demonstrating a maximum at the T2 solution and unique solutions for Equation **??** in our ROI. The vertical lines show the T2 at the example voxel for each ROI.

Table S1. Full table of tissue volumes for all the subjects.

Table S2. Full table of measurements for regions of interest.

How to cite this article: S. Bhattacharya, A. Price, A. Uus, H. S. Sousa, M. Marenzana, K. Colford, P. Murkin, M. Lee, L. Cordero-Grande, R. P. A. Gomes Teixeira, S. J. Malik, and M. Deprez (2023), In-vivo T2 measurements of fetal brain Using Single-Shot Fast Spin Echo Sequences, *Magn. Reson. Med.*, *2023;In progress.*

TABLE S1 Full table of tissue volumes for all the subjects.

Tissue / Erosion → / GA (weeks)↓	Cortex (Tissue volumes (mL))		White Matter		Deep Grey Matter		Subplate	Total Brain volume (mL)
	Not eroded	Eroded	Not eroded	Eroded	Not eroded	Eroded	Not Eroded	
**21 Weeks**	12.22	6.32	23.12	18.18	1.79	1.38	9.37	76.55
**27 Weeks**	33.92	20.29	83.71	74.08	7.40	6.21	29.83	228.60
**29 Weeks**	24.51	14.13	54.51	47.19	5.50	4.55	18.99	144.30
**31 Weeks**	44.54	27.84	95.05	83.89	8.51	7.22	27.15	205.86
**35 Weeks**	73.92	48.49	123.53	106.68	11.50	9.94	30.03	299.53

This table provides the volumes of each tissue ROI comparing the eroded and non-eroded volumes where applicable.

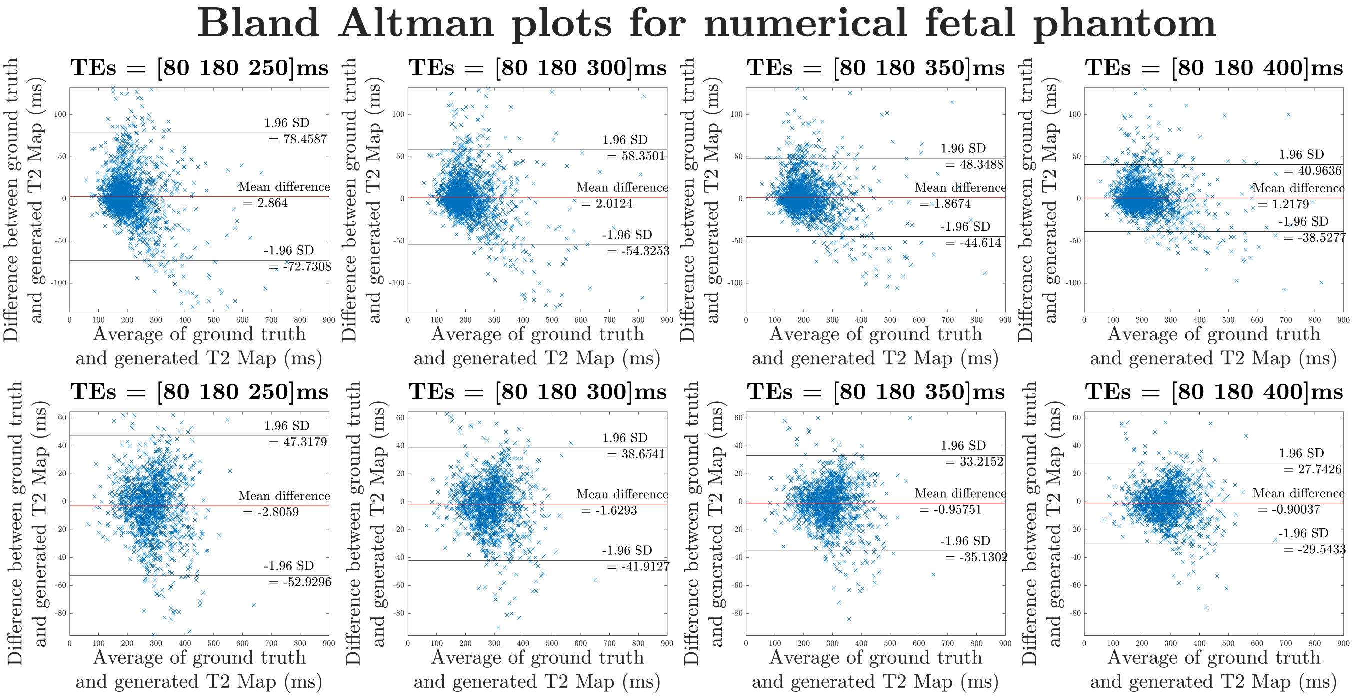

FIGURE S1 Bland-Altman plots for the numerical phantom measurements to demonstrate the optimal sequences used. The top row is the Bland-Altman plots for the cortical and DGM and the bottom row is the WM.

TABLE S2 Full table of measurements for regions of interest

Tissue	Fetal T2 (ms) $\pm\sigma$ (ms) GA = 21 weeks	GA = 27 weeks	GA = 29 weeks	GA = 31 weeks	GA = 35 weeks	Mean
**Cortex**	250 ± 58	206 ± 65	167 ± 52	200 ± 44	172 ± 47	199 ± 33
**DGM**	211 ± 28	237 ± 41	185 ± 26	205 ± 28	169 ± 28	201 ± 26
**WM**	285 ± 76	334 ± 68	237 ± 43	310 ± 58	253 ± 55	283 ± 39
**Subplate**	307 ± 65	343 ± 69	246 ± 41	305 ± 50	258 ± 56	292 ± 40

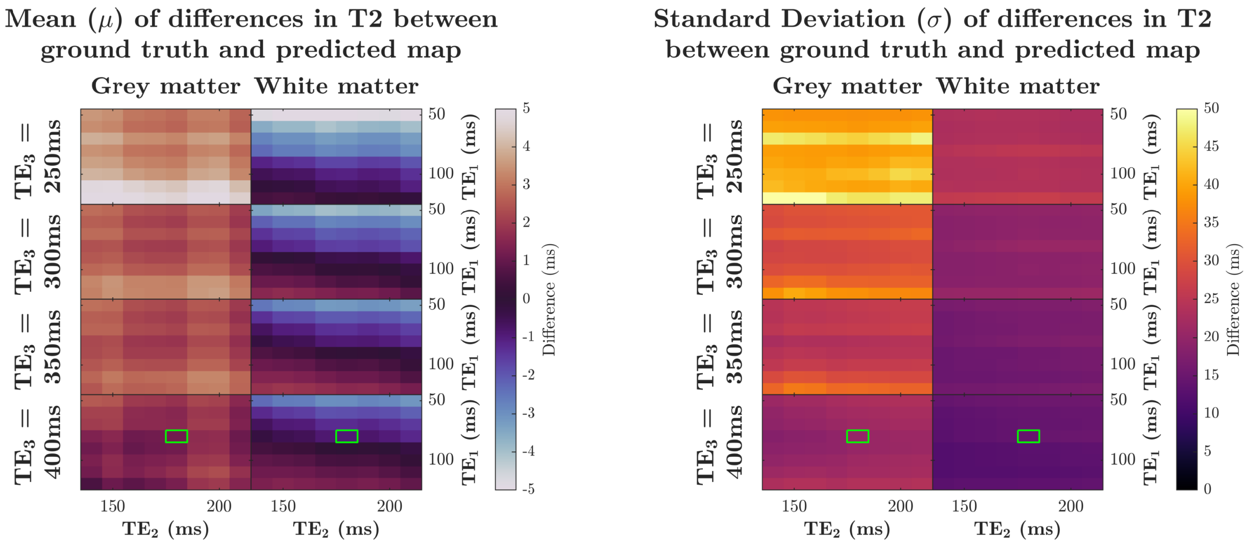

FIGURE S2 Heatmaps showing the effects of all three TEs on the difference between the T2 values of ground and predicted map. TE_2 does not significantly impact the prediction, TE_1 slightly affects the mean difference (or bias) and TE_3 significantly affects both the mean differences and the standard deviation of differences. Our chosen sequences are highlighted with a green rectangle

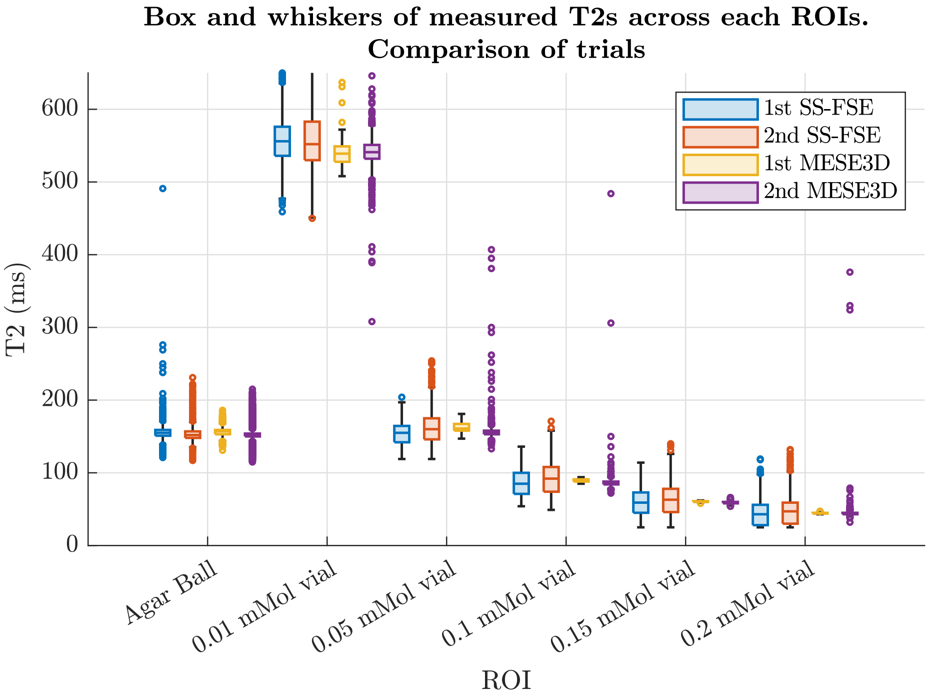

FIGURE S3 Plots showing the comparison of T2 results from two sessions to assess repeatability of the measurements. These show repeated overlap with of all T2s; both the standard MESE and the proposed SS-FSE sequences.

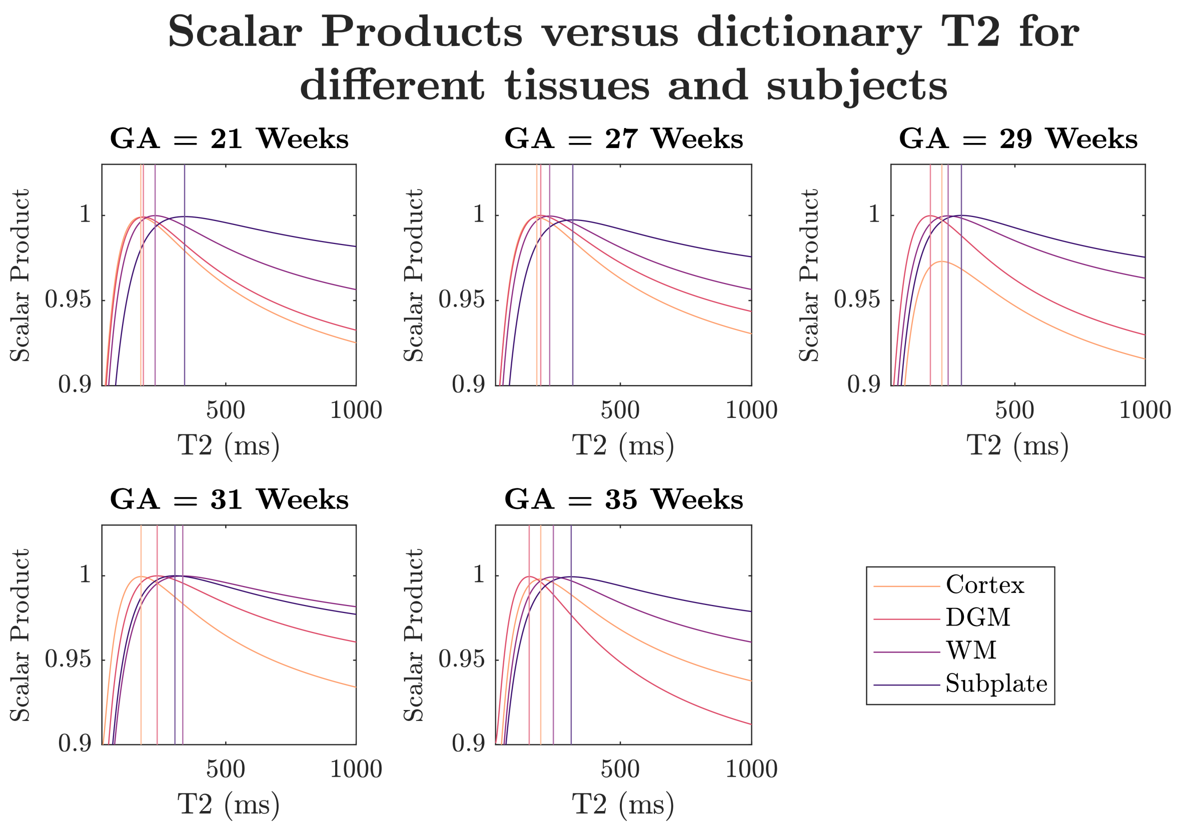

FIGURE S4 Plots showing Scalar products for a voxel within the given ROI, demonstrating a maximum at the T2 solution and unique solutions for Equation **??** in our ROI. The vertical lines show the T2 at the example voxel for each ROI.
